# Molecular defects in the mannose binding lectin pathway in dermatological disease: Case report and literature review

**DOI:** 10.1186/1476-7961-8-6

**Published:** 2010-03-25

**Authors:** Christopher Miller, Sara Wilgenbusch, Mini Michael, David S Chi, George Youngberg, Guha Krishnaswamy

**Affiliations:** 1 From the Departments of Pathology, Quillen College of Medicine, East Tennessee State University, Johnson City, TN 37614, USA; 2 Internal Medicine, Quillen College of Medicine, East Tennessee State University, Johnson City, TN 37614, USA; 3 Division of Allergy Clinical Immunology, Quillen College of Medicine, East Tennessee State University, Johnson City, TN 37614, USA; 4 The James H. Quillen VA Medical Center, Mountain Home, TN, USA

## Abstract

Mannose-binding lectin (MBL) and the Mannose-binding lectin-associated serine proteases (MASPs) are an essential aspect of innate immune responses that probably play an important but understudied role in cutaneous function. The MBL-MASP pathway appears to exert its primary role by assisting in the clearance of apoptotic skin cells (thus preventing accumulation and a subsequent autoimmune response) and promoting opsonophagocytosis of invading pathogens, limiting their dissemination. Deficiencies of the pathway have been described and are associated with infectious, autoimmune and vascular complications. However, the role of this pathway in dermatological disease is essentially unexplored. We describe 6 patients presenting with recurrent inflammatory and/or infectious skin conditions who also demonstrated severely low MBL levels. One patient also had a defect in the MASP2 gene. Genotype analysis revealed specific point mutations in the *MBL2 *promoter in all 6 patients and a variant MASP-2 gene in one patient. Five patients presented recurrent pustular skin infections (cellulitis, folliculitis and cutaneous abscess). A case of Grover's disease and one forme fruste of Behcet's syndrome (orogenital ulcers) were also observed. The patients responded to antimicrobial therapy, although in some, recurrence of infection was the rule. It appears that MBL deficiency may contribute to recurrent skin infections and to certain forms of inflammatory skin disease. The mechanisms may relate to the role of this pathway in innate immunity, removal of apoptotic cells and in immune complexes. Further study of MBL pathway defects in dermatological disease is required.

## Introduction

The skin represents the largest organ of the innate immune system, composing not only a physical barrier but also containing numerous elements important in the immunological response against invading pathogens (e.g. keratinocytes, macrophages, Langerhans cells, dendritic cells, dermal fibroblasts). Damage to this barrier predisposes the body to a more susceptible environment for microbial dissemination, while improper immune surveillance can be a triggering factor for several inflammatory skin diseases [1]. This is an intricately orchestrated defense system constituted by a local response at the level of the epidermis and dermis, as well as by systemic involvement, with migration of additional immune cells to the site of antigenic stimulus.

**Figure 1:**
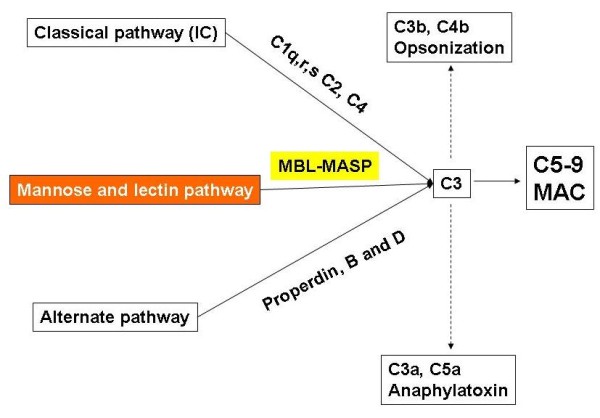
**Schematic representation of the three complement activation pathways, converging to cleave C3**. Classical pathway (activated by immune complexes), MBL-pathway (activated by bacterial sugars such as mannose) and the alternate pathway (activated even in the absence of antibody), result in formation of the C3 convertase, which culminates in C3 activation. After this occurs, C3b will opsonize the surface of the pathogen, with subsequent phagocytosis. Further progression of the cascade leads to the activation of C5-9 and to the formation of the membrane attack complex (MAC), lysing the microbe/cell. Byproducts of these pathways include anaphylotoxins C3a and C5a, recruiting leukocytes which contribute to the inflammatory response.

A member of the collectin group of pattern recognition receptors, mannose-binding lectin (MBL) is part of the innate immune system, a primordial defense mechanism that serves as the initial response to host invasion by pathogens in an antibody-independent fashion (Figure 1). This is achieved through direct opsonization of bacteria, recruitment of phagocytic cells that promote phagocytosis of pathogens, along with complement activation and immunomodulatory cytokine production that promote chemotaxis and recruitment of inflammatory cells, thereby limiting pathogenic spread. Defective MBL production is regarded as the most common immune deficiency in the general population, affecting approximately 5-7% of individuals [2], although some descriptions have delineated higher figures among Caucasians (up to 30%) [3]. The implications of low MBL levels have been the target of a large volume of research, with an unequivocal influence on host susceptibility to a variety of recurrent infectious processes and autoimmune disorders. However, propensity to dermatological disease has not been explored to any great extent. One report using MBL-deficient mice demonstrated upregulation of inflammatory cytokines and chemokines, thinning of the dermis and epidermis, as well as eschar separation, in response to burn injury [4]. Other investigators have suggested that diseases such as atopic dermatitis, that present defects in innate immunity, may have a defective MBL response [5]. Nevertheless, the role of MBL-MASP pathway defects in dermatological disease is sparse, and we believe our report to be the first to link MBL deficiency to recurrent infectious and inflammatory skin disease in 6 patients. Further studies in this area are obviously sorely required. This is especially important as treatment with recombinant MBL may soon be available and may assist some patients with otherwise refractory or serious dermatological disease.

## Case Presentation

**Figure 2:**
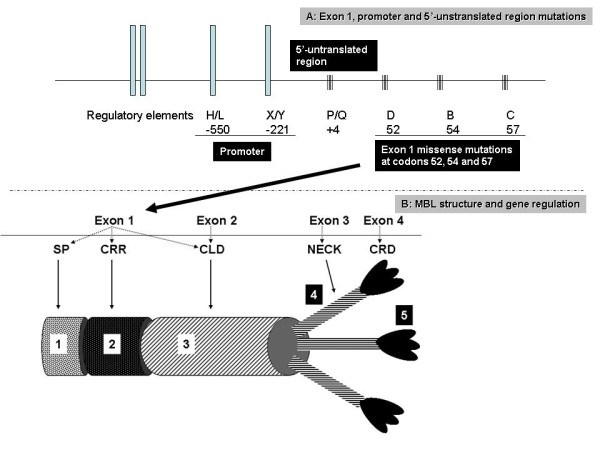
**Molecular genetic and structural aspects of MBL**. Representation of the gene organization of *MBL2 *(MBL1 is a pseudogene)and the locations within Exon 1 that most commonly present polymorphisms, resulting in the variant alleles B, C, and D (codons 54, 57, and 52, respectively), in addition to the P/Q variant at position +4 (5'-Unstranslated region). Upstream from Exon 1, the sites of promoter mutations that lead to the H/L and X/Y variants are also demonstrated. The *MBL2 *gene is comprised of four exons. Exon 1 encodes the serine protease domain (SP) (1), the cysteine-rich region (CRR) (2), and a portion of the collagen-like domain (CLD) (3); exon 2 encodes the remainder of the CLD; exon 3 encodes the neck region (4); and exon 4 encodes the carbohydrate-recognition domain (CRD) (5). Components of the MBL gene that process transcription of these various components of the fully assembled MBL protein are also designated in this cartoon. Location of insert (A) showing mutations on Exon map is represented by black arrow.

The study was approved by the Institutional Review Board (East Tennessee State University) and the Research and Development Committee of the James H. Quillen Veterans Affairs Medical Center in Mountain Home, Tennessee. The records of the patients were reviewed and appropriate data collected. Immunoglobulin assays were carried out in commercial laboratories by traditional techniques. MBL genotyping and functional assays were carried out by the IBT laboratories, Lenexa, Kansas. MBL genotypes were assigned as per Figure 2. Tables 1 and 2 list the laboratory results and immunological evaluation in these patients.

### Patient 1

A 39-year-old woman complained of recurrent pustular eruption of her upper extremities and chronic allergic rhinitis. Immunological evaluation demonstrated the following: IgG levels were slightly decreased, but IgG subclass levels were all normal. The patient had detectable/robust responses to 6/14 pneumococcal serotypes (Table 1). Further work-up yielded a low level of MBL at < 50 ng/ml (reference: >100 ng/ml), as well as an impaired MBL pathway functional test using the C4b deposition assay. Genotyping showed the HYPD/HYPD variant of *MBL2 *and wild-type MASP-2 genotype (A/A) (Table 2).

**Table 1:** Outline of additional immune deficiencies encountered in the patient group

Patient	Laboratory assay	Patient value	Reference range
#1	Total immunoglobulin G (IgG)	768 mg/dL	791-1643 mg/dL
	IgG subclasses	Normal	
	Robust response to pneumococcal serotype	6/14	> 8/14
	Functional MBL pathway (C3b deposition assay)	2.02	> 5.1
#2	Immunoglobulin and complement levels	Normal	
	Functional MBL pathway (C3b deposition assay)	2.22	> 5.1
#3	C4 level	Mild deficiency (1 null allele out of 4)	4 functioning alleles
	Functional MBL pathway (C3b deposition assay)	2.04	> 5.1
#4	Immunoglobulin and complement levels	Normal	
	C-reactive protein	10 mg/L	< 6 mg/L
	Antinuclear antibody	1/8 (weakly positive)	< 1/8
	Functional MBL pathway (C3b deposition assay)	2.10	> 5.1
#5	Immunoglobulin and complement levels	Normal	
	Functional MBL pathway (C3b deposition assay)	2.18	> 5.1
#6	IgG3	34 mg/dL	41-129 mg/dL
	Functional MBL pathway (C3b deposition assay)	Not available	

**Table 2:** Summary of clinical scenarios and respective genotyping results

Patient	Clinical condition	*MBL2*	MASP-2
#1	Rhinoconjunctivitis and recurrent upper extremity skin infections	HYPD/HYPD	A/A
#2	History of MRSA pacemaker infection and recurrent folliculitis	LYPB/HYPD	A/A
#3	Recurrent *S. aureus *folliculitis	LYPB/HYPD	A/A
#4	Fungal folliculitis	LXPA/LYPB	A/G
#5	Forme fruste of Behçet's disease	LXPA/LYPB	A/A
#6	Recurrent lower extremity cellulitis and ulcerations	LYPB/LYPB	A/A

### Patient 2

**Figure 3:**
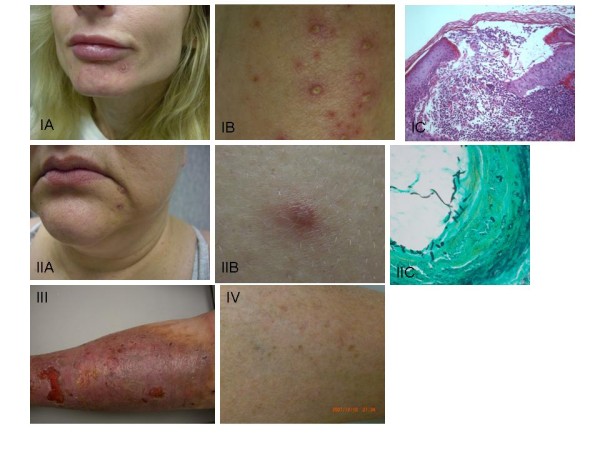
**Patients with MBL deficiency and dermatological disease**. Figures I A, B and C demonstrates pustular skin eruption, with biopsy showing neutrophilic inflammation in patient #3. Figures II A, B and C demonstrate fungal folliculitis with PAS stain (C), demonstrating fungal hyphal elements in patient #4. Figure III shows lower extremity cellulitis and inflammatory ulcer of patient #6, and figure IV demonstrates folliculitis-like rash of patient #2 with Grover's Disease and history of recurrent staphylococcal skin infections.

A 61-year-old man presented with recurrent folliculitis-like eruptions of the skin. His history was significant for a prior history of infection of the cheek with methicillin-resistant *Staphylococcus aureus *(MRSA) treated with minocycline. Six years earlier, he had suffered infection of a pacemaker site with MRSA, which had spread to his upper extremities, but was ultimately controlled with antibiotics. He denied intravenous drug use. Physical examination revealed a 1-cm indurated nodule over his left cheek, a 5 × 3 cm hyperpigmented patch over his left ankle, and scattered areas of erythema over his anterior chest wall and dorsum of his legs. MBL level was < 50 ng/ml, while all classes of immunoglobulins were within normal range (Table 1). MBL genotyping revealed the LYPB/HYPD haplotypes. MASP-2 genotype was the wild type (A/A). MBL functional pathway test was impaired (Table 2). A skin biopsy of the folliculitis-like rash (Figure 3, IV) was performed, revealing Grover's disease (transient acantholytic dermatosis). Topical therapy was instituted with triamcinolone.

### Patient 3

A 47-year-old woman presented with a pustular rash (Figure 3, I A, B and 3, I C) on her skin (face, back, forearms and leg) and pustular inguinal eruptions; on one occasion she had developed a vulvar infection with abscess formation requiring drainage. Past medical history included recurrent rhinosinusitis and anxiety disorder. Biopsy of the lesion showed pustular changes with neutrophilic infiltration, but did not reveal lymphocyte infiltration. A culture from a lesion on her forearm was positive for *Staphylococcus aureus*. The patient responded to administration of cefadroxil and minocycline, with remission and frequent relapses. Laboratory workup revealed mild C4 deficiency and an undetectable IgE level (Table 1). Assays were unremarkable for ANA, ESR, rheumatoid factor, hepatitis viruses, and HIV. The MBL level was exceptionally low at < 50 ng/ml, and her MBL functional pathway was impaired. MBL genotyping revealed a LYPB/HYPD haplotype, while MASP-2 was wild-type (Table 2).

### Patient 4

49-year-old woman with complaints of a blistering skin eruption and hyperpigmentation for the past 6 years; the lesions were thick, tender, indurated, and presented with yellow-green secretion. Her past medical history included recurrent impetigo, perennial rhinitis, and ulcerative colitis. On exam, a 1-inch area of induration and erythema was observed over her chin (Figure 3II A and 3II B); there were multiple scattered pustular lesions over the forearms, some ulcerated. Encrusted pustules were also observed over her scalp, face, back and legs. Work-up yielded a weakly positive ANA and an elevated CRP (10 mg/L) (Table 1). MBL levels were low at < 50 ng/ml. Punch biopsy from her forearm (Figure 3, II C) revealed histopathology and morphology compatible with *Candida *sphaerica, although the possibility of *Trichophyton rubrum *was also raised; cultures were attempted from a specimen but no growth was observed. An aspirate of one of the pustules grew *Sphingomonas paucimobilis*. Terbinafine and levofloxacin were given, and her eruption resolved. Genotyping revealed LXPA/LYPB for MBL and A/G for MASP-2 (Table 2). The MBL functional assay was also impaired.

### Patient 5

A 48-year-old woman presented with painful orogenital ulcerations. She had a family history of documented Behcet's disease (BD) in her father and brother and she was concerned she may have the disease. She was uncertain as to whether she was of Melungeon descent (peoples of Tri-racial descent who have lived in the South-Eastern United States: these include European, African/Middle Eastern/Turkish and Native American). Pertinent other history included severe polyarthralgia (hands, wrists, arms, knees, and ankles) over the past 8 years.

A pathergy test was negative. Immunoglobulin levels, ESR, and CRP were unremarkable. Assays for hepatitis viruses, HIV, rheumatoid factor, Herpes simplex, and lupus anticoagulant were negative (Table 1). Ophthalmologic evaluation did not demonstrate the characteristic uveitis. She was treated with a pulse of oral glucocorticoids and placed on long term hydroxychloroquin. After 5 months, oral and genital ulcerations had resolved. Due to the clinical response to therapy but insufficient diagnostic criteria, she was diagnosed with a forme fruste of BD. Further work-up established an MBL level < 50 ng/ml and a deficient MBL functional pathway (Table 1). Genotyping revealed an LXPA/LYPB haplotype for MBL and A/A for MASP-2 (Table 2).

### Patient 6

A 56-year-old Caucasian man complained of recurrent bilateral lower extremity cellulitis and ulcerations, requiring hospitalization and parenteral antibiotic administration. History included factor V Leiden mutation with recurrent lower extremity deep venous thromboses and recurrent MRSA urinary tract infections. He had undergone numerous abdominal procedures, with recurrent enterocutaneous fistulae and wall abscesses. Abdominal exam revealed wall ulceration with serosanguineous drainage and surrounding erythema. His lower extremities presented ochre dermatitis, bilateral +/4 edema below the knee; there were 2 ulcerations on the anterior portion of his left leg, measuring 1 × 1 cm and 2 × 3 cm (Figure 3, III). A lower extremity ultrasound showed a left-sided nonocclusive chronic thrombus in the femoral and popliteal veins. Total levels of immunoglobulins were normal (except for a small decrease in IgG3), as were C2 and C4 (Table 1). Anticardiolipin antibody was negative. The patient had a low level of MBL (<50 ng/ml), genotyping showing a LYPB/LYPB polymorphism for MBL and a wild-type MASP-2 (Table 2). The patient's abdominal wall culture grew cephalosporin-sensitive *Staphylococcus aureus*; cultures from his lower extremity ulcerations were positive for *Pseudomonas aeruginosa*. The patient responded to antimicrobials, compression stockings and topical Tacrolimus^©^.

## Disscussion

The MBL-MASP pathway of complement activation results in multiple beneficial effects in the innate response to pathogens. Deficiencies of the MBL-MASP pathway have been linked to recurrent and serious infections, atopic disease, and autoimmunity, as well as to cardiovascular pathology. However, the role of this pathway in dermatological disease has not been studied adequately.

The circulating MBL-2 protein is an oligomeric molecule formed by three identical polypeptides which spiral around each other in a tight helix to form a subunit, several of which link together to compose higher-order oligomers (Figure 2). It is through the carbohydrate-recognition domain (CRD) that pathogen-associated molecular patterns (PAMPs) are recognized and bound, with biological activity occurring through clustering of CRDs and multiple carbohydrate binding to the widely-spaced, repetitive sugars on the surface of pathogens [6-8]. Upon binding to PAMPs, the MBL pathway and MASP recruitment are activated. MASP-2 is analogous to C1s as it forms C3 convertase by sequentially cleaving C4 and C2. Deposition of C3b on the microbial cell surface is the common point between the 3 complement pathways, after which either opsonophagocytosis will occur or the membrane attack complex will form pores in the cell wall and lead to osmotic lysis (Figure 1).

There are three functional single nucleotide polymorphisms within exon 1 of the *MBL2 *gene which lead to low serum MBL levels. The A allele is also termed "wild-type" and is usually associated with normal MBL levels. The variant alleles (B, C and D) are collectively termed O alleles and confer a distorted molecular structure, as well as low serum MBL. There are seven *MBL2 *haplotypes, 4 of which are associated with decreased MBL levels (LYPB, LYQC, HYPD, and LXPA), the other 3 (HYPA, LYPA, and LYQA) producing normal levels [9]. Serum MBL will be about one-fifth of normal range in heterozygotes and less than 2% in homozygotes or compound heterozygotes [10].

Alternative splicing and polyadenylation of the *MASP-2/MAp19 *gene lead to encoding of MASP-2 and MAp19. Recent reports have identified a mutation in exon 3 of this gene leading to the exchange of aspartate with glycine at position 105 (D105G) within the CUB1 domain, causing impaired binding to MBL, and also low circulating plasma levels of MASP-2 [11,12]. Wild-type genotypes (AA) present with normal serum levels of MASP-2, while homozygotes for the mutation (GG) will display little or no lectin pathway activity. Heterozygous genotypes (AG) confer approximately half the amount of circulating MASP-2.

The patient in ***Case 1 ***manifested recurrent upper respiratory tract infections, most likely of an occupational nature, in addition to repeated skin findings. The exact nature of her skin condition was not clearly elucidated, but the fact that she presented a good response to antihistamine and steroid therapy suggests atopy, reinforced by concomitant respiratory tract involvement. The association between recurrent upper respiratory infections and MBL deficiency has been grounds for debate by a number of studies [13-16]. MBL promotes complement-mediated killing of a number of upper respiratory pathogens [17,18]. In the case of pneumococcus, MBL has an ancillary part in promoting opsonophagocytosis, and low serum levels have been associated with increased mortality [19]. Our patient displayed mild deficiency in total IgG with normal subclasses, manifest through a diminished antipneumococcal immunoglobulin production, which may occur in the absence of a specific subclass deficiency [20].

In ***Case 2***, the development of MRSA infection at the site of pacemaker placement may have been a trigger for recurrent skin findings, with several failed attempts at eradicating the organism. MBL participates in first-line defense against *S. aureus *via opsonophagocytosis by macrophages and neutrophils [21]. The MBL-MASP complex has a significant role in increasing C3b deposition on the surface of *S. aureus *[22], contributing to an increase in its engulfment by neutrophils. In addition, C3b combines with factor B to generate the alternative pathway convertase (C3bBb), which promotes further breakdown of C3b [23]. Thus, the lectin and alternative pathways have developed mutual amplification loops [24], although experimental evidence suggests that the alternative pathway has a much greater influence on controlling staphylococcal bacteremia than the lectin or classical pathway [25]. In addition, MBL triggers macrophage release of cytokines in response to bacterial invasion, enhancing neutrophilic activity. MBL is a modulator of inflammation, interacting with toll-like receptors in NF-κB-dependent production of TNF-α; the latter is a crucial element in host defense, and its deficiency impairs recruitment of neutrophils and phagocytosis [26]. MBL-null mice incubated with intravenous *S. aureus *had a blunted production of TNF-α and interleukin-6 (IL-6) reversible by pretreatment with recombinant human MBL (rhMBL) [21]. The influence of the C4 deficiency in our third patient is questionable, highlighted by a murine study by Cunnion et al. showing similar mortality from bacteremia caused by encapsulated *S. aureus *as compared to controls with normal C4 [25].

A potentially confounding factor in ***Case 2 ***is the concurrent diagnosis of Grover's disease, which has been associated with immunodeficiency [27]. Although usually self-limited, one-third of patients may be refractory to therapy. Of note, success in treating this condition with antibiotics or antimycotics should raise questions about the veracity of the diagnosis [27].

The role of MBL deficiency in recurrent fungal folliculitis is not entirely clear, but it is likely that fungal mannan may be able to activate the complement cascade that then leads to activation of a phagocytic and cellular immune response. Defense against dermatophyte infection relies greatly on cell-mediated immunity to destroy the stratum corneum in which the pathogens reside (seen as how complement and humoral elements have limited access to this layer), aided by a Th1-type delayed hypersensitivity reaction [28-31]. MBL has been shown to have a strong binding affinity for *Candida albicans *and acts in first-line defense against this organism, increasing C3 deposition on its surface and hence phagocytosis by neutrophils [32,33].

Due to the diagnosis of BD in 2 other family members, a possible familial clustering was suggested in ***Case 5***, as this disease has a strong genetic influence and is distributed within certain geographic locations (i.e. Mediterranean and Asia) [34,35]. Unfortunately, an accurate familial background was not provided by the patient. A potential role for microorganisms (e.g. streptococci, viruses) has been suggested in the etiopathogenesis of BD, due to a hyperactive response to bacterial antigens and/or heat shock proteins in an already proinflammatory state [36,37]. Hence, innate immunity would be essential in limiting invasion and an exaggerated immune response, with MBL deficiency representing a susceptibility and severity factor in BD [37]. Apart from impairing local defenses (potentially facilitating recurrent oral ulcers), low MBL levels allows a more constant pathogenic stimulation, leading to immune cell infiltration and a Th1-type cytokine profile (with increased levels of IL-12 and interferon-γ) [36,37]. In addition, MBL has an important role in clearance of apoptotic cells (MBL is recruited from noncutaneous sources to bind apoptotic keratinocytes and induce their uptake by dendritic cells) [38], the accumulation of which can favor autoimmunity, as the release of intracellular components triggers inflammatory cytokine production and development of reactive clones, leading to autoantibody formation [39,40]. In the specific case of BD, there are large numbers of PMNs located within mucocutaneous lesions, and MBL deficiency contributes to impaired clearance of these cells after they undergo apoptosis, precipitating the immune response described [37]. Of note, the negative pathergy test (intradermal saline injection looking for a granulomatous reaction at the injection site) displayed by our patient does not exclude the diagnosis, as it is only positive in around 30% of cases.

In addition to recurrent infections with *S*. *aureus *and *P*. *aeruginosa*, against which MBL plays a role in primary defense, the patient in ***Case 6 ***presented with complicated postoperative courses. Postoperative infections have already been shown to occur at a greater rate in MBL-deficient patients (haplotype A/O, O/O in exon 1 and AX/AX), and preoperative serum levels are actually a form of identifying patients at risk [41,42]. Now, while MBL deficiency has been shown to predispose to arterial thrombosis in patients with autoimmune disorders, no increased risk for development of venous thrombosis was demonstrated [43]. But, in this particular case, his hypercoagulable condition is the most likely culprit.

The major limitation in our study is the small number of cases, the lack of appropriate controls and the high frequency of MBL deficiency in the population. This might suggest that these findings are coincidental and that MBL deficiency may have no relationship to the infections experienced by the reported group of patients. Nevertheless, we feel this report is among the first to connect recurrent and sometimes serious skin infections and inflammatory conditions to the MBL-MASP pathway. We hope this report will lead others to look for these defects. Finally, this series of case reports could lead to a better-designed study to evaluate the prevalence and significance of MBL-MASP pathway defects in patients with cutaneous infections. Also of note, it is likely that in a subset of patients, MBL deficiency may need to be accompanied by yet another immune defect, such as in the classical complement, humoral immune or phagocytic pathways to manifest infection (such as has been demonstrated in patients with chemotherapy-related infection). The pathogenic role of MBL-MASP pathway in infectious and inflammatory dermatoses needs to be defined and more studies are required.

A number of our patients would be likely candidates for MBL replacement therapy, particularly those who did not display a satisfactory response to conventional therapies. Several trials have met with promising results in Europe, but use of recombinant human MBL (rhMBL) in the United States is not yet approved. Recently, a second-generation plasma-derived MBL (pMBL) product has been manufactured and is undergoing phase II trials. Work with stem cell preparations has given ground to new hypotheses suggesting possible cell-signaling properties inherent to MBL replacement therapy, which may include induction of immunologic maturation in autologous cells, in addition to simply increasing the serum levels for the extent of half-life of the product [44].

## Conclusions

The MBL-MASP pathway of complement activation is of importance to innate immunity and to response to pathogens. While defects in the pathway have been linked to infectious, vascular and autoimmune disease, no prior reports have stressed the importance of these defects to dermatological disease. We present six cases who had profound defects in the MBL-MASP pathway and who developed serious cutaneous infectious and/or inflammatory disease. It appears that MBL deficiency may contribute to recurrent skin infections and to certain forms of inflammatory skin disease. The mechanisms may relate to the role of this pathway in innate immunity, removal of apoptotic cells and immune complexes. Further study of MBL pathway defects in dermatological disease is required as it may open up treatment options with recombinant MBL protein (when available) for patients with difficult-to-treat dermatological infections.

## Abbreviations

MBL: mannose-binding lectin; rhMBL: recombinant MBL; pMBL: plasma-derived MBL; MASP: MBL-associated serine protease; ANA: antinuclear antibody; ESR: erythrocyte sedimentation rate; HIV: human immune deficiency virus; BD: Behcet's disease; MRSA: methicillin-resistant staphylococcus aureus; IgG: immunoglobulin G; PAMP: pathogen-associated molecular patterns; CRP: C reactive protein; CRD: carbohydrate recognition domain; NF-κB: nuclear factor kappaB; TNF-α: tumor necrosis factor alpha; C4: complement component 4; IL-6: interleukin-6; PMN: polymorphonuclear leukocyte.

## Competing interests

The authors declare that they have no competing interests.

## Authors' contributions

CM carried out some literature search and partially drafted the manuscript. SW helped to draft part of the manuscript and proofread the manuscript. MM helped to collect and analyze the data. DSC helped to draft the manuscript, revised it for important intellectual content, and assisted the finalizing of the manuscript. GY was responsible for patient data and analysis. GK conceived and managed the study, drafted the manuscript, managed references, generated figures and tables, and has given final approval of the version to be published. All authors have read and approved the final manuscript.

## Written Consent

Written informed consent was obtained from the patients for publication of this case report and accompanying images. A copy of the written consent is available for review by the Editor-in-chief of this journal.
